# Beyond FITT: addressing *density* in understanding the dose–response relationships of physical activity with health—an example based on brain health

**DOI:** 10.1007/s00421-025-05858-3

**Published:** 2025-06-26

**Authors:** Fabian Herold, Liye Zou, Paula Theobald, Patrick Manser, Ryan S. Falck, Qian Yu, Teresa Liu-Ambrose, Arthur F. Kramer, Kirk I. Erickson, Boris Cheval, Yanxia Chen, Matthew Heath, Zhihao Zhang, Toru Ishihara, Keita Kamijo, Soichi Ando, Joseph T. Costello, Mats Hallgren, David Moreau, Vahid Farrahi, David A. Raichlen, Emmanuel Stamatakis, Michael J. Wheeler, Neville Owen, Sebastian Ludyga, Henning Budde, Thomas Gronwald

**Affiliations:** 1 Department of Physiology, Faculty of Medicine, HMU Health and Medical University Erfurt, Erfurt, Germany; 2 Institute of Interdisciplinary Exercise Science and Sports Medicine, MSH Medical School Hamburg, Am Kaiserkai 1, 20457 Hamburg, Germany; 3 Research Group Degenerative and Chronic Diseases, Movement, Faculty of Health Sciences Brandenburg, University of Potsdam, Potsdam, Germany; 4 Body-Brain-Mind Laboratory, Shenzhen University, Shenzhen, China; 5 Motor Control and Learning Group, Department of Health Sciences and Technology, Institute of Human Movement Sciences and Sport, ETH Zurich, Zurich, Switzerland; 6 Division of Physiotherapy, Department of Neurobiology, Care Sciences and Society, Karolinska Institute, Stockholm, Sweden; 7 School of Biomedical Engineering, The University of British Columbia, Vancouver, BC Canada; 8 Aging, Mobility, and Cognitive Health Laboratory, Department of Physical Therapy, The University of British Columbia, Vancouver, BC Canada; 9 Djavad Mowafaghian Centre for Brain Health, Vancouver Coastal Health Research Institute, Vancouver, BC Canada; 10 Centre for Aging Solutions for Mobility, Activity, Rehabilitation and Technology (SMART) at Vancouver Coastal Health, Vancouver Coastal Health Research Institute, Vancouver, BC Canada; 11 Center for Cognitive and Brain Health, Northeastern University, Boston, MA USA; 12 Department of Psychology, Northeastern University, Boston, MA USA; 13 Beckman Institute for Advanced Science and Technology, University of Illinois at Urbana-Champaign, Urbana, IL USA; 14 Department of Neuroscience, AdventHealth Research Institute, AdventHealth, Orlando, FL USA; 15 Department of Psychology, University of Pittsburgh, Pittsburgh, PA USA; 16 Center for the Neural Basis of Cognition, University of Pittsburgh, Pittsburgh, PA USA; 17 Department of Sport Sciences and Physical Education, Ecole Normale Supérieure Rennes, Bruz, France; 18 Laboratory VIPS2, University of Rennes, Rennes, France; 19 School of Kinesiology, Faculty of Health Sciences, University of Western Ontario, London, ON N6A 3K7 Canada; 20 Canadian Centre for Activity and Aging, University of Western Ontario, London, ON N6A 3K7 Canada; 21 Graduate Program in Neuroscience, University of Western Ontario, London, ON N6A 3K7 Canada; 22 Graduate School of Human Development and Environment, Kobe University, Kobe, Japan; 23 Faculty of Liberal Arts and Sciences, Chukyo University, Nagoya, Japan; 24 Graduate School of Informatics and Engineering, The University of Electro-Communications, Tokyo, Japan; 25 Extreme Environments Laboratory, School of Psychology, Sport and Health Sciences, University of Portsmouth, Portsmouth, UK; 26 Epidemiology of Psychiatric Conditions, Substance Use and Social Environment (EPiCSS), Department of Public Health Sciences, Karolinska Institute, Solna, Sweden; 27 Institute for Physical Activity and Nutrition (IPAN), School of Exercise and Nutrition Sciences, Deakin University, Geelong, Australia; 28 School of Psychology and Centre for Brain Research, University of Auckland, Auckland, New Zealand; 29 Institute for Sport and Sport Science, TU Dortmund University, Dortmund, Germany; 30 Human and Evolutionary Biology Section, Department of Biological Sciences, University of Southern California, Los Angeles, CA 90089 USA; 31 Department of Anthropology, University of Southern California, Los Angeles, CA 90089 USA; 32 Mackenzie Wearables Research Hub, Charles Perkins Centre, University of Sydney, Sydney, NSW Australia; 33 Faculty of Medicine and Health, School of Health Sciences, University of Sydney, Sydney, NSW Australia; 34 Physical Activity Laboratory, Baker Heart & Diabetes Institute, Melbourne, VIC Australia; 35 School of Health Sciences, Swinburne University of Technology, Melbourne, VIC Australia; 36 Department of Sport, Exercise and Health, University of Basel, Basel, Switzerland; 37 Institute for Systems Medicine (ISM), MSH Medical School Hamburg, Hamburg, Germany; 38 G-Lab, Faculty of Applied Sport Sciences and Personality, BSP Business and Law School, Berlin, Germany

**Keywords:** Physical exercise, Sedentary behavior, Brain, Cognition, Dose

## Abstract

Research on physical activity (PA) and health has a fundamental concern with dose–response relationships. The variables of (1) Frequency, (2) Intensity, (3) Time, and (4) Type (i.e., the FITT principle) have traditionally been used to operationalize the dosage of PA. We consider some limitations of FITT and propose that it can be complemented by the additional variable *density* (from the German exercise and training variable *Belastungsdichte),* which can be defined as the timing of successive work bouts within a single PA bout as well as the timing between successive PA bouts within a specific time period; it does so by quantifying the temporal intervals between successive work or PA bouts (i.e., time spent at a lower PA intensity or resting such as in napping/sleeping or sedentary behaviors). Using the field of PA and brain health as an example, we discuss the opportunities and challenges for further research employing the variable *density* and consider its potential to improve the understanding of dose–response relationships between PA and health outcomes.

## Introduction

Physical activity (PA), which includes planned and structured forms of acute and chronic physical exercise (see Table 1 for definition), is widely recognized as an important factor in maintaining and improving overall health (Bull et al. 2020; Warburton and Bredin 2017; World Health Organization 2020, 2022). However, the optimal dosage of PA, including but not limited to the time point at which PA should be initiated or repeated to trigger changes in specific measures of health, is not well understood (Bull et al. 2020; World Health Organization 2020). In this context, there is currently a need for greater clarity in the definition of the dosage of PA (Gronwald et al. 2018b, 2019, 2020b; Herold et al. 2019a, 2020c; Impellizzeri et al. 2023), with a call for a more complete reporting of dosage in intervention studies using PA (Hansford et al. 2022; Bland et al. 2021; Solis-Urra et al. 2024; Gronwald et al. 2019). From a practical perspective, elucidating the complex dose–response relationships of PA with health outcomes, including intra- and inter-individual response variability, is an important prerequisite when aiming to maximize the benefits of PA interventions by individualizing PA prescription (Barha et al. 2017b, 2021a, b; Herold et al. 2019a, 2021; Solis-Urra et al. 2024).

**Table 1:** Definition of key terms

Key terms	
Brain health	…is defined as the optimal development and maintenance of brain integrity and encompasses: (1) structural (e.g., hippocampal volume) and functional (e.g., changes in brain activity) brain parameters; (2) functions that depend on the integrity of the brain, including but not limited to mental health, cognition, and movement; and (3) the absence of neurological disorders (e.g., dementia) (Wang et al. 2020; Zou et al. 2024).
Dose / dosage	…is characterized by three key components: (1) external load (i.e., defined as the work performed by the individual independent of internal characteristics), (2) influencing factors (i.e., all factors [e.g., including environmental factors] that can strengthen or weaken the stimuli of a single bout of PA), and (3) internal load (i.e., defined as the individual and acute physiological, psychological, motor, and biomechanical responses to the external load and the influencing factors during and/or after the cessation of a single bout of PA). Thus, the dose can be determined and monitored by using specific indicators of internal load involved in the biological processes that drive the desired changes in outcomes of interest (Gronwald et al. 2020b; Herold et al. 2019a, 2020b). The term *dose* characterizes the PA stimuli *at a specific time*, whereas the term *dosage* refers to the *dose provided over a particular time* (Sharma and Dunham 2024).
Physical activity (PA)	…is defined as any voluntary skeletal muscle-induced bodily movement (e.g., in occupational or leisure time) that results in an increase in the energy expenditure (Bull et al. 2020; Caspersen et al. 1985; World Health Organization 2020, 2022) above ∼1.5 metabolic equivalents of the task (MET; 1 MET = 1 kcal (4.184 kJ) kg^−1^ h^−1^) (Budde et al. 2016; Falck et al. 2021; Herold et al. 2018a, 2022b; Zou et al. 2024). This includes planned and structured forms such as acute and chronic physical exercise (see the following definition). PA can be divided into acute (single bout/session) and chronic (multiple bout/session) PA based on temporal characteristics (Budde et al. 2016; Herold et al. 2018a, 2022b). Furthermore, PA can be differentiated based on the domains in which it occurs, including recreation/leisure time (such as household), transportation, education, or occupation (Bull et al. 2020; Dipietro et al. 2020; Falck et al. 2021; Howley 2001; Pettee Gabriel et al. 2012; Strath et al. 2013; World Health Organization 2020).
Physical exercise	…is defined as a specific form of PA that is planned and structured (Budde et al. 2016; Caspersen et al. 1985; Herold et al. 2018a, 2022b; World Health Organization 2020, 2022). Exercise can be divided into acute (single bout/session) and chronic (multiple bouts/sessions) based on temporal characteristics, also referred to as physical training when it is repetitive, and designed to improve or at least maintain the performance in one or more fitness dimensions (Budde et al. 2016; Caspersen et al. 1985; Herold et al. 2018a, 2022b; Howley 2001; World Health Organization 2020, 2022). In addition, physical exercise is typically performed in recreational/leisure time when it is not part of healthcare service (e.g., rehabilitation) or occupation (e.g., elite athlete). Notably, physical exercise is always PA, whereas PA is not necessarily physical exercise (Wegner et al. 2020).
Sedentary behavior (SB)	…is defined as any waking behavior characterized by a low energy expenditure (≤ 1.5 MET) while in a sitting, reclining, or lying posture (Bull et al. 2020; Falck et al. 2021; Sedentary Behaviour Research Network 2012; Tremblay et al. 2017; World Health Organization 2020, 2022). SB is ubiquitous, due to rapid changes in human environmental, economic, social, and technological contexts. SB has been identified as a newer component of the activity spectrum that can adversely impact health (Dunstan et al. 2021, 2012; Katzmarzyk et al. 2019; Pinto et al. 2023). SB can be categorized as mentally active (e.g., reading and computer use) and mentally passive (e.g., watching non-educational television programs) (Zou et al. 2024; Hallgren et al. 2020). For many adolescents and adults, the daily time spent SB is ≥ 5 h (Bauman et al. 2011; Raichlen et al. 2023; Yang et al. 2019).”

*PA* physical activity, *MET* metabolic equivalent of the task, *SB* sedentary behavior

The dose of PA traditionally has been characterized and prescribed using the FITT principle, an acronym representing: (1) *Frequency* (i.e., number of PA bouts in a specific time interval), (2) *Intensity* (i.e., level of exertion required by an individual to perform PA), (3) *Time* (i.e., duration spent for PA), and (4) *Type of PA* (i.e., specific kind of PA such as endurance, resistance or coordinative activities) (Festa et al. 2023; Hecksteden et al. 2018; Heisz and Waddington 2024; Li et al. 2024; Liguori et al. 2022; Noone et al. 2024; Oberg 2007; Zubin Maslov et al. 2018). The FITT principle can also be used to analyze how the dosage of free-living PA (e.g., unplanned and unstructured forms of PA) is associated with health-related outcomes. Some researchers have suggested extending the FITT principle to FITT-VP (Liguori et al. 2022; Bushman 2018) by including the factors of: (5) *Volume* (V), which is typically provided as a product of the frequency and duration of the acute PA bouts (e.g., total PA or PA spent in a particular intensity zone) (Assis et al. 2018; Di Liegro et al. 2019; Hecksteden et al. 2018); and, (6) *Progression* (P), which characterizes the gradual and systematic increase of the PA stimulus to maintain overload and, thus, provoke further adaptation(s) to overall physical and brain health (Kasper 2019).

Although adhering to the FITT and the FITT-VP principles can help standardize the variables that should be considered for PA prescription and dose–response analysis, this approach has several limitations. First, neither FITT nor FITT-VP takes into account all acute and chronic variables (e.g., movement frequency) that determine the dosage of PA (especially of planned and structured forms such as acute and chronic physical exercise) (Gronwald et al. 2020b; Herold et al. 2019a, 2020c; Toigo and Boutellier 2006). In addition, they provide an overall framework that includes a mixture of variables to prescribe PA (i.e., frequency, intensity, time, type, and volume) and one selected training principle (i.e., progression). Second, FITT-VP does not consider the timing of PA stimuli within a specific time interval, namely the temporal interval between successive bouts of PA, which is quantified by the time spent resting and conceptualized as density (see also definition below, (Desgorces et al. 2020; Hernández-Lougedo et al. 2021; Herold et al. 2019a, b, 2025; Manci et al. 2024; Törpel et al. 2018). Third, each component of the FITT and FITT-VP principle is treated somewhat independently when in fact the variables characterizing PA are interdependent (Gronwald et al. 2020b; Toigo and Boutellier 2006). Indeed, intensity is significantly influenced by other variables such as acute duration (Hofmann and Tschakert 2017; Tschakert et al. 2022) and movement frequency (e.g., cadence measured as revolutions per minute when using a cycle ergometer) (Beneke and Leithäuser 2017; Gronwald et al. 2018a).

As illustrated by consideration of the aforementioned limitations of FITT and FITT-VP, determining or providing a specific dosage of PA is complex, and the oversimplification of PA dosage may hinder the accurate prediction and optimization of PA interventions on health (Gronwald et al. 2020b; Herold et al. 2019a, 2020c). As we will show, considering the variable *density* provides a more subtle approach that goes beyond FITT and FITT-VP (Herold et al. 2019a, 2025; Manci et al. 2024) and allows for a more precise determination of the PA dosage (Gronwald et al. 2020b; Herold et al. 2019a, 2020c; Manci et al. 2024). A more precise determination of PA dosage can, in turn, advance our understanding of the dose–response relationships between PA and health-related outcomes (Gronwald et al. 2020b; Herold et al. 2019a, 2020c).

To provide the reader with a concrete idea of how density can enrich our understanding of dose–response relationships between PA and health-related outcomes, we use brain health as an example (see Table 1 for the definition of key terms). Our rationale to focus on a single example is twofold. First, the mechanisms mediating the effects of PA on health-related outcomes are diverse, and dose–response relationships can depend on the specific endpoint (Warburton and Bredin 2016). Thus, it was necessary to focus on a particular endpoint. Second, disorders affecting brain health (e.g., dementia) are a growing public health concern (Gustavsson et al. 2023; Nichols et al. 2022; Velandia et al. 2022; Wimo et al. 2023) with evidence that the reduction of modifiable risk factors, such as physical inactivity, can be a cost-effective countermeasure (Livingston et al. 2024; Norton et al. 2014; World Health Organization 2019). Our focus on brain health is therefore intended to provide a broadly applicable, albeit sufficiently specific, example of the relevance of PA density to health-related outcomes. Furthermore, summarizing the current state of evidence concerning the role of density for other health-related outcomes (e.g., cardiovascular, metabolic, musculoskeletal) or intervention approaches (e.g., cognitive training, hypoxia and/or heat training/therapy) is beyond the scope of this article, although we acknowledge that the variable density is also relevant for those applications.

## Method

The German exercise and training variable “Belastungsdichte” (Hottenrott et al. 2022, Schnabel et al. 2014) (hereafter referred to as *density*), which has its roots in the field of exercise science, is not well-recognized internationally, and is only occasionally mentioned in the literature (Desgorces et al. 2020; Hernández-Lougedo et al. 2021). Thus, we seek to improve its accessibility by introducing this variable to the broader exercise- and health-science community. In this context, we also extend the description and application of density to the field of free-living PA (a key concern for public health), where it has not previously been applied. Indicated by the fact that density is largely absent in the literature (e.g., in work reporting (Herold et al. 2019b; Manser et al. 2024), analyzing (Chang et al. 2012; Falck et al. 2019; Kao et al. 2022; Ludyga et al. 2020; Oberste et al. 2019, 2021; Pontifex et al. 2019), or providing recommendations on PA (World Health Organization 2019) in the context of brain health) and thus underappreciated in the exercise-science and health research communities, we opted to perform a narrative review because there is probably not a sufficiently large and specific evidence base to conduct a systematic review (e.g., on the role of density of PA on brain health). The author group comprises an international mix of junior, mid-career, and senior researchers from different disciplines, and cultural and ethnic backgrounds who have provided critical feedback about the conceptualization and theoretical position that we describe here.

## Definition of density

Density characterizes the timing of PA (also referred to as *work bout*[*s*]) within a single PA bout as well as the timing between successive PA bouts within a specific time period (e.g., day, week, month, or year); it does so by providing information on the temporal intervals (i.e., time spent resting; also referred to as *rest, recovery,* or *relief bouts*) within or between successive PA bouts (Desgorces et al. 2020; Herold et al. 2019a, b, 2025; Hernández-Lougedo et al. 2021; Manci et al. 2024; Törpel et al. 2018).

From a mechanistic perspective, the effect of density on a dependent variable (e.g., a specific health-related outcome such as brain health) can be studied by keeping the characteristics of work bouts similar (i.e., in terms of acute and chronic variables that characterize PA) while modifying the duration of rest bouts. In other words, density can be modified by changing the duration of the rest bout(s) to adjust the work–rest ratio.

**Fig. 1:**
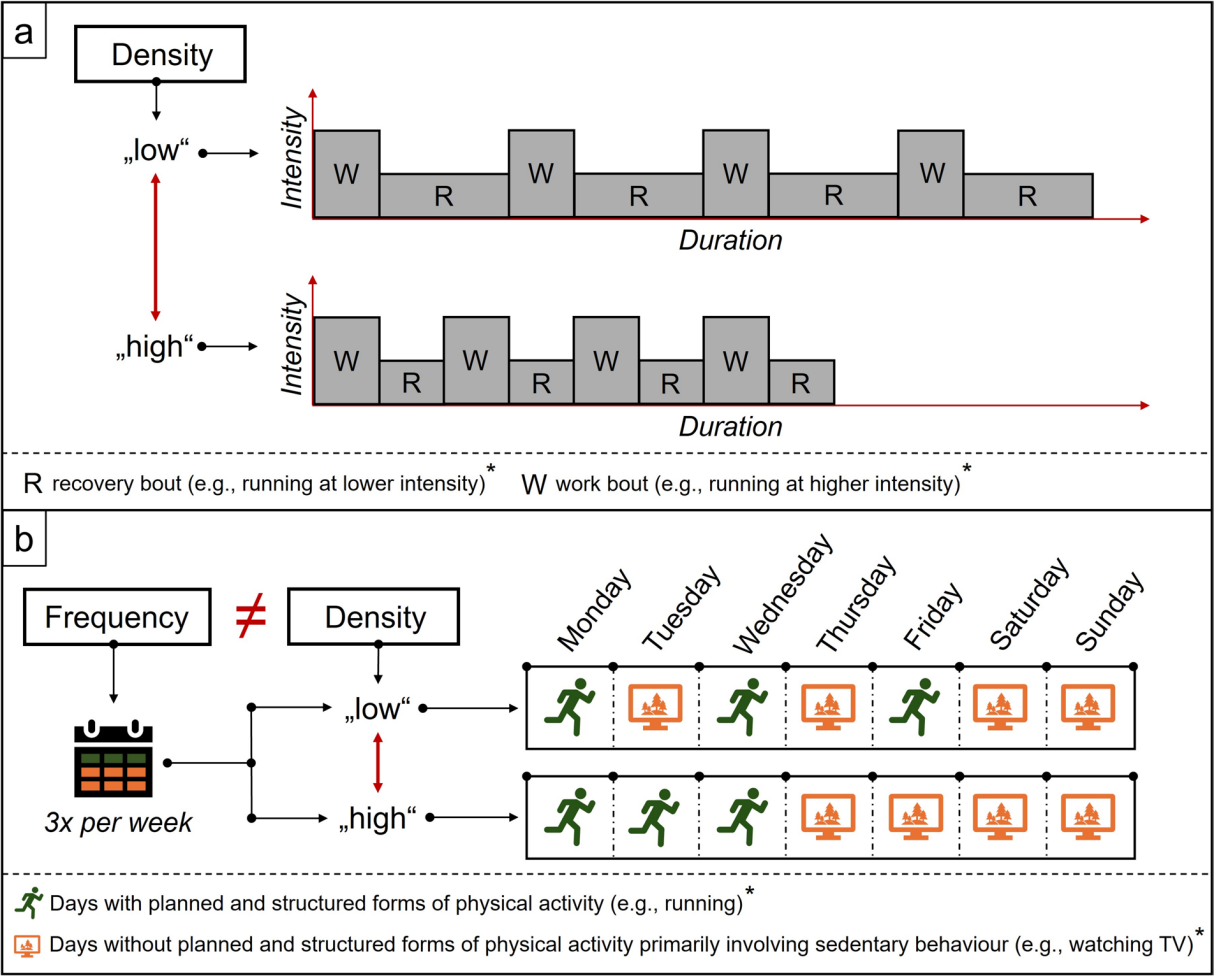
Panel (**a**) upper: Schematic illustration of different densities, using an acute bout of physical exercise in interval mode as an example. Here, the number of the work bouts (4x) and rest bouts (4x) is equal whereas the duration of the rest bout in the upper panel (i.e., low acute density; the work–rest ratio of 1:2) is twice as long as in the lower panel (high acute density; the work–rest ratio of 1:1) resulting in a different acute density and, in turn, physical activity dosage. In this example, an active rest bout, which is conducted at half the intensity of the work bout, is selected. The example also illustrates that specific acute variables are interrelated (e.g., acute density, acute duration, and intensity of work and rest intervals). Panel (**b**) lower: Schematic illustration of the difference between frequency and chronic density in the context of chronic physical activity. The visualization shows that the same frequency (3× physical activity bouts per week) can be distributed differently over a week, resulting in a different chronic density and, in turn, physical activity dosage. The asterisk (*) indicates that other acute (i.e., type of physical activity, intensity, and acute duration) and chronic variables (i.e., chronic duration) that characterize the bout(s) of physical activity are assumed to be constant. Please note that we used sedentary behavior as an example for the rest bout(s) in panel B; however, depending on the context, such rest bout(s) can also encompass physical activity at a lower intensity than that of the work bout(s). Further, quantifying chronic density also depends on the period of interest (e.g., day, week, month, year)

In this context, we highlight three important points. First, as shown in Table 2, density is conceptually different from existing concepts and variables (e.g., frequency, rest-activity rhythm (RAR), volume, work–rest ratio, interruptions to sedentary time) that are relevant to prescribing the PA dosage, and has a significant potential to complement approaches to analyze and promote PA. Second, based on the temporal context of PA, density needs to be further differentiated into acute density (i.e., in the context of variations within single shorter bouts of PA; see Fig. 1 a) and chronic density (i.e., in the context of repetitions of PA over longer periods; see Fig. 1b) (Gronwald et al. 2020b). Third, given that the key element defining density is the rest bout, it is also worth noting that in addition to rest duration, there are other variables, especially the type of activity (e.g., sedentary behavior (SB) or sleep), that need to be considered when prescribing or analyzing PA because they can moderate the effect of PA on brain health (see also Sect. "Chronic density — Sophisticated analysis approaches" and "Recommendations regarding the assessment of chronic density").

**Table 2:** Overview of differences and synergies between density with other selected physical activity-related concepts. *PA* physical activity, *RAR* rest-activity rhythm, *VILPA* vigorous intermittent lifestyle physical activity

Variable/ concept	Relation to density
*Differences between density and other selected PA-related concepts*	
Physical activity (PA) variability	Density differs from the metric of PA variability (i.e., the standard deviation of minute-to-minute changes in device-based PA counts across a particular wear period; Donahue et al. 2025) because it quantifies the rest duration between successive PA bouts instead of the variability of PA patterns.
Frequency	Density captures information beyond that provided by frequency because frequency only specifies the number of PA bouts in a given time interval (e.g., day, week, month, year) (Bushman 2018; Garber et al. 2011; Hecksteden et al. 2018; Herold et al. 2019a) but not their timing *within* that interval (e.g., rest duration between successive PA bouts).
(Circadian) Rest-activity rhythm	Density has some conceptual overlap but differs from the construct of (circadian) rest-activity rhythm (RAR). Although both constructs deal with PA and rest patterns, density provides information on the time intervals between successive PA bouts within a specific time period because it provides information on the exact rest duration. In contrast, the construct of (circadian) RAR primarily focuses on investigating regularity, fragmentation, and amplitude of the rest-activity rhythms across different time frames (e.g., biological rhythms such as sleep–wake cycle throughout periods ≥ 24 h) (Calogiuri et al. 2013; Gao et al. 2023; Keihani et al. 2023; Smagula 2016; Smagula et al. 2019). In other words, density is more concerned with defining the precise rest duration between successive bouts of PA (Buchheit and Laursen 2013b; Desgorces et al. 2020; Herold et al. 2019a, b, 2025; Hernández-Lougedo et al. 2021; Manci et al. 2024; Törpel et al. 2018) while the metrics used to operationalize (circadian) RAR typically describe rest-activity patterns and their variability (Danilevicz et al. 2024a, 2024b; Gao et al. 2023). Furthermore, density can be used to analyze and prescribe PA (Herold et al. 2025; Manci et al. 2024), while RAR has only been used to analyze PA patterns (Danilevicz et al. 2024a, b; Gao et al. 2023; Smagula 2016).
Volume	Density differs from volume, which is typically described as the product of the frequency and duration of acute PA bouts (e.g., of total PA or PA spent in a particular intensity zone) (Assis et al. 2018; Di Liegro et al. 2019; Hecksteden et al. 2018) because volume lacks information on the exact temporal interval(s) between successive PA bouts *within* a specific time period.
Weekend warrior PA pattern	Density has some conceptual overlap but differs from the weekend warrior approach because a “weekend warrior” PA pattern is typically characterized by achieving a specific amount of PA (e.g., recommended by the World Health Organization such as ≥ 150 min of moderate- or ≥ 75 min of vigorous-intensity PA per week (World Health Organization 2020, 2022)) in ≤ 2× bouts per week (Hamer et al. 2017; Lee et al. 2004; Min et al. 2024; Ning et al. 2024, 2025; O'Donovan et al. 2018; Wu et al. 2024) without taking the temporal interval(s) (i.e., rest duration) between those PA bouts into account, which is the key feature of density.
Work–rest-ratio	Density is associated with changing the time spent at rest (i.e., duration of the rest/recovery/relief bout[s]), whereas the work–rest ratio can also be adjusted by changing the duration of the work bout(s) (Buchheit and Laursen 2013b).
*Potential synergies of density and other selected PA-related concepts*	
Physical activity paradox	Density, defining the temporal intervals between successive PA bouts within a specific time period with greater precision, may help to explain the “PA paradox” that occupational PA has less clear or no health benefits compared to leisure-time PA (Holtermann et al. 2012, 2018, 2021b; Pronk 2024; Stamatakis et al. 2024). This phenomenon is perhaps related to the fact that a key distinction can be made between active and passive (sedentary) occupations (Owen et al. 2020) because active professions (e.g., construction workers, or farmers) often perform substantial occupational PA at higher intensities in relatively short time intervals (i.e., higher acute density) compared to others (e.g., office workers) (Owen et al. 2020). In this context, density may also help to identify “sweet spots” to individualize leisure-time PA recommendations by considering occupational PA levels (Holtermann et al. 2021a).
Time of day	Density can add the concept of and time of day (e.g., specifying whether PA is conducted in the morning, afternoon, or evening (Bruggisser et al. 2023; Ingham-Hill et al. 2024; Janssen et al. 2022; Sabag et al. 2024)) by providing more precise information on the temporal intervals between successive PA bouts within a specific time period (i.e., by quantifying the exact rest duration between PA bouts performed at a specific time of day).
VILPA or exercise snacks	Density, as a characteristic defining the PA dosage, can help to more precisely elucidate the influence of different rest bout durations between short work bouts of physical activity (e.g., conducted at vigorous intensity or other intensities in the context of free-living PA such as light- or moderate-intensity PA) on health-related outcomes. When such short work bouts are performed at a vigorous intensity, they are conceptualized in the *vigorous intermittent lifestyle physical activity* (*VILPA*) approach, which is defined as brief vigorous-intensity bouts of incidental PA occurring during daily living (e.g., fast stair-climbing) and lasting up to 1 or 2 min (Ahmadi et al. 2024; Stamatakis et al. 2022, 2023; Thøgersen-Ntoumani et al. 2024), and “*exercise snacks*” approach, which are defined loosely as single planned bouts of physical exercise that typically (1) lasts ≤ 1 min, (2) occurs multiple times throughout the day, and (3) are performed at a vigorous intensity (Islam et al. 2022; Stamatakis et al. 2020; Wang et al. 2024).

## Density is important—but neglected—when investigating dose–response relationships between physical activity and health

In the current literature on PA and brain health, the FITT principle is also commonly used when analyzing the dose–response relationship between PA and measures of brain health (Cabral et al. 2019; Falck et al. 2019; Gallardo-Gómez et al. 2022; Northey et al. 2018), but the nature of the evidence examined indicates that: (1) acute density is typically not considered when analyzing the influence of acute bouts of PA on cognitive performance (i.e., as a moderator variable) (Chang et al. 2012; Kao et al. 2022; Oberste et al. 2019, 2021; Pontifex et al. 2019), (2) chronic density is often not reported in studies investigating the influence of chronic PA on cognition (Herold et al. 2019b; Manser et al. 2024), (3) chronic density is absent in moderator analyses in recent systematic reviews and meta-analyses investigating the influence of chronic PA on cognitive performance (Falck et al. 2019; Ludyga et al. 2020), and (4) chronic density is typically not mentioned in recommendations (e.g., from the World Health Organization) and policies aimed at reducing the risk of cognitive decline and dementia by lifestyle changes (e.g., via PA) (World Health Organization 2019). The absence of the variable density in the literature, in analyses of dose–response relationships, and in the recommendations of official bodies could lead to the assumption that acute and chronic density are unimportant variables or that researchers studying the effects of PA on brain health may be unaware of the importance of density.

Given that neither the precise PA dosage (Bherer et al. 2013; Falck et al. 2019; Ludyga et al. 2020; Stillman et al. 2020) nor the neurobiological mechanisms that mediate the positive effects of acute and chronic PA on brain health are fully understood (Herold et al. 2018b, 2020a; Hou et al. 2024; McMorris 2021; Pontifex et al. 2019; Stillman et al. 2020; Voss 2016; Zou et al. 2023), density is a promising candidate for advancing our understanding in this direction. Specifically, considering density will allow for a more precise definition of the minimal and optimal PA dosage by providing information on how the duration of the rest or sedentary bout(s) between PA stimuli within a single bout of PA or successive bouts of PA (i.e., work bouts) influence specific measures of brain health. Generating such evidence holds great potential to inform and optimize intervention approaches aimed at promoting PA (e.g. “just-in-time adaptive PA interventions” (Hardeman et al. 2019; Müller et al. 2017; Wunsch et al. 2022)) to break up prolonged sitting with acute bouts of PA (Chueh et al. 2022; Li et al. 2022; Wheeler et al. 2019; Yu et al. 2022).

To illustrate how accounting for density can be crucial in the context of PA and brain health, we outline below several lines of evidence supporting our assumption. In this context, we highlight further directions for observational and intervention studies.

### Acute density

A recent study provided direct evidence that acute density can influence the acute PA-related effects on specific behavioral measures of brain health. In particular, this study used a within-subject crossover design with a pretest–posttest comparison to investigate in healthy younger adults whether the use of different inter-set rest intervals (i.e., 1 min versus 3 min, representing higher and lower acute densities) during an acute bout of low-load resistance exercise (i.e., 40% of a one-repetition maximum, 6× sets of 10× repetitions) can influence acute exercise-induced changes in inhibitory control (i.e., assessed with the Stroop test) (Tomoo et al. 2021). In this study, shorter inter-set rest intervals (i.e., 1 min—higher acute density) improved inhibitory control (i.e., operationalized by a reverse Stroop interference score) immediately, 10 min, 20 min, and 30 min after exercise cessation, whereas such effects were absent for longer inter-set rest intervals (i.e., 3 min—lower acute density) (Tomoo et al. 2021). These findings support the notion that considering acute PA density has great potential to advance the understanding of the dose–response relationship of acute PA on specific measures of brain health.

### Chronic density

There is a growing interest in the scientific community in developing a more holistic understanding of the chronic influence of the 24-h activity cycle (Collins et al. 2023; Mellow et al. 2019, 2022) and the distribution of PA across a defined time interval (e.g., including but not limited to concepts such as “weekend warrior” (Min et al. 2024; Ning et al. 2025; O'Donovan et al. 2024; Wu et al. 2024), time of day (Maeneja et al. 2022), or fragmentation (Marino et al. 2024, 2025; Wanigatunga et al. 2022)) on brain health. In the following sections, we show that chronic PA density is a candidate determinant of brain health effects that should not be overlooked in observational and intervention studies, and when analyzing dose–response relationships within the context of chronic PA-related benefits on specific measures of brain health.

#### Observational studies

Five observational studies analyzed population-based data in adults, regarding the role of achieving a specific amount of PA recommended by the World Health Organization (i.e., 50% (Min et al. 2024; Ning et al. 2024, 2025) or 100% (Wu et al. 2024) of ≥ 150 min of moderate- or ≥ 75 min of vigorous-intensity PA per week (World Health Organization 2020, 2022) in ≤ 2× bouts per week (i.e., denoted as “weekend warrior”) or ≥ 3× bouts per week on cognition and risk of developing chronic disease that affect brain health (Min et al. 2024; Ning et al. 2025; Wu et al. 2024).

In one of these observational studies, middle-aged to older adults with a weekend warrior PA pattern exhibited, regardless of the daily SB duration, a lower dementia risk compared to physically inactive adults with a long daily SB duration (i.e., < 150 min of moderate-to-vigorous-intensity PA and ≥ 8.5 h of SB) (Ning et al. 2025). Furthermore, another study reported that adults with a weekend warrior PA pattern showed a reduced risk of disorders that negatively affect brain health (i.e., all-cause dementia and Parkinson’s disease) compared to those being physically inactive (i.e., < 150 min of moderate-to-vigorous-intensity PA) (Ning et al. 2024), with evidence that adults with a such an PA pattern had a comparable risk reduction for such disorders (e.g., dementia, stroke, Parkinson’s disease, depressive or anxiety disorder) compared to those engaging more often in PA (i.e., ≥ 3× PA bouts per week) (Min et al. 2024). The latter observation is consistent with the findings of another prospective study in Mexican adults that showed that compared to those who did not engage in planned and structured forms of leisure-time PA, “weekend warriors” (i.e., ≤ 2× PA bouts per week) had a comparable risk reduction for mild dementia (i.e., operationalized by a score in the Mini-Mental Status Test of ≤ 22 points) relative to those who regularly engaged in PA (≥ 3× PA bouts per week) (O'Donovan et al. 2024). In the other studies, healthy adults with a weekend warrior and non-weekend warrior PA pattern showed comparable benefits in the cognitive composite score relative to those who were physically inactive (i.e., < 150 min of moderate-to-vigorous-intensity PA), whereas in adults with depressive symptoms, only those engaging in a non-weekend warrior PA pattern showed statistically significant improvements in cognitive functions (Wu et al. 2024).

Although neither of those five observational studies considered chronic density — because they did not account for the rest duration between the successive bouts of PA — several studies indicated that healthy adults achieving the recommended amount of PA in ≤ 2× bouts per week had a comparable positive influence on cognitive performance and dementia risk as achieving this amount of PA in ≥ 3× bouts per week. Whether such observations extend to other measures of brain health when the moderating role of the chronic density of PA is considered is an important area for further investigation. However, the finding that among individuals with depressive symptoms, only those with a non-weekend warrior PA pattern benefited in terms of cognition (Wu et al. 2024), supports the idea that considering chronic PA density is an important variable to gain insights into mechanisms explaining this observation.

#### Intervention studies

Although one 15-week intervention study in healthy older adults did not observe an effect of manipulating PA distribution via a modification of the work bout (i.e., performing multicomponent exercise for 30 min in the morning and the afternoon versus 60 min in the morning) on behavioral measures of brain health (i.e., executive functions) (Monteagudo et al. 2021), to the best of our knowledge, no intervention study has explicitly focused on modifying the chronic density of a PA intervention to investigate its causal influence on brain health. However, a meta-analysis suggests that a higher frequency (i.e., 5–7× PA bouts per week), which is typically achieved by exercising almost or every day in a week (i.e., probably mirrored in a high chronic density due to short rest duration, when assuming that commonly only 1× PA session is conducted per day), is more beneficial for improving cognitive performance in adults older than 50 years (i.e., double the effect size; 0.69 versus 0.32) than a lower frequency (i.e., 1–2× PA bouts per week) (Northey et al. 2018). Given that remains open whether such an observation might be related to a higher density or a higher total volume of PA, future intervention studies that purposefully manipulate chronic density or at least appropriately report acute and chronic density are needed to broaden the evidence base on the influence of different PA densities on brain health.

In this context, providing information on acute and chronic density can be especially relevant for interventions with lower levels of direct supervision (e.g., home- and technology-based interventions using exergames). For example, in home-based studies using exergames and providing only general supervision, partial direct supervision, or even no supervision [for more information on supervision please see (Denton et al. 2021; Herold et al. 2024a, b)], older adults are typically instructed to achieve a certain duration of physical exercise over a week but are often allowed to self-select the frequency of the acute PA bouts (Callisaya et al. 2021; Delbaere et al. 2015; Gschwind et al. 2015a, b; Hoang et al. 2016; Schoene et al. 2013, 2015; Song et al. 2018). Such studies have documented that older participants who are highly motivated can exceed the recommended training frequency and/or perform multiple PA bouts throughout the day (Gschwind et al. 2015a; Hoang et al. 2016; Manser and Bruin 2024; Manser et al. 2023). This may result in insufficient rest time, which is perhaps less than optimal for the materialization of adaptation processes (i.e., consolidation). The above theoretical assumption is supported by (1) an experimental study showing that in younger adults too much consecutive computer-based training can be detrimental to learning performance (i.e., accuracy of motion discrimination) (Ashley and Pearson 2012) and (2) a systematic review observing that cognitive performance declines when endurance athletes are overtrained (Symons et al. 2023). These latter findings support the assumption that acute and chronic exercise density should be considered when prescribing and monitoring interventions aimed at promoting brain health.

Acute and chronic density are important variables in the prescription of physical exercise (i.e., periodization, and programming of sessions) because they further characterize the dosage by defining the duration of rest bout(s) within a single bout of physical exercise or between successive bouts of physical exercise (i.e., work bouts). Whereas periodization is the temporal organization (i.e., macro-management) of the characteristics of physical exercise sessions and phases of training (e.g., purposeful adjustment of variables such as exercise intensity and volume for progression) and application of training principles (Brown and Greenwood 2005; Cunanan et al. 2018; Kataoka et al. 2021; Ratamess et al. 2009), programming is defined as the micro-management of physical exercise that includes but is not limited to the organization of exercise and training variables (e.g., type of physical exercise, exercise intensity, exercise duration, and acute and chronic density) (Cunanan et al. 2018; Herold et al. 2019a; Kataoka et al. 2021). Thus, acute density is especially relevant for programming single physical exercise sessions within physical training, wherein physical exercises are performed in an interval mode or a set structure. This is because acute density defines the rest duration between the work bouts (e.g., also referred to as intervals or repetitions), between interval series or sets, or between different physical exercises (Buchheit and Laursen 2013a, b; Ratamess et al. 2009). As shown in Fig. 1, the acute PA stimulus can be modified by decreasing or increasing the duration of rest between successive work bouts (i.e., higher or lower acute PA density, respectively).

## Assessment and prescription of acute and chronic density

In the following sections, we discuss different prescription and analysis approaches for PA density considering the temporal context, the availability and accessibility of population-based datasets, and recent technological advances to assess PA (i.e., miniaturized wearables to track lifestyle activities within the 24-h activity cycle). Typically, the operationalization of chronic PA density becomes more challenging when longer time intervals are considered (e.g., week, month, year), especially for unplanned and unstructured forms of PA. Thus, we describe simple and more sophisticated analysis approaches relating to the chronic density of PA.

### Acute density

Within a single session of PA, acute density can be operationalized by the duration of the rest bout(s) between the successive work bouts (i.e., in seconds or minutes or relative to the duration of the work bout; see Fig. 1a). Thus, a modification of acute PA density can be achieved by decreasing or increasing the duration of the rest bout(s), resulting in a higher acute work–rest ratio (i.e., higher PA density) or a lower acute work–rest ratio (i.e., lower PA density), respectively.

### Recommendations concerning the assessment and prescription of acute density

As we will demonstrate below, temporal dynamics on different levels of analysis (e.g., behavioral, functional brain, and molecular and cellular levels (Erickson et al. 2022; Stillman et al. 2016, 2020)), particularly the after-effects of PA, are important sources of evidence to inform the prescription of acute PA density.

On the behavioral level, two meta-analyses showed that the after-effects of acute physical exercise on cognitive performance are transient, depending on the characteristics of the physical exercise, such as type, intensity, and duration (Chang et al. 2012; Garrett et al. 2024). More specifically, according to these meta-analyses, the greatest effects of acute physical exercise on cognitive performance can be expected to be 11–20 min (Chang et al. 2012) or 20–75 min (Garrett et al. 2024) after the cessation of the acute physical exercise bout and diminishes with longer delays (Chang et al. 2012; Garrett et al. 2024). However, some studies provide evidence that the after-effects of acute physical exercise on specific cognitive domains (e.g., executive functions) can even persist for up to 60 min in children (Ludyga et al. 2019) and up to 30 to 90 min in healthy younger adults (Dora et al. 2021; Hung et al. 2013; Martínez-Díaz and Carrasco Páez 2023; Tian et al. 2021; Tomoo et al. 2020; Tsukamoto et al. 2016a). Moreover, in healthy younger adults, performing acute physical exercise four hours after learning is more beneficial for improving memory performance and hippocampal pattern similarity (i.e., assessed 48 h later) than performing acute physical exercise immediately after learning the task (van Dongen et al. 2016).

The above evidence suggests that the effects of acute PA on cognitive performance are transient. Thus, it seems reasonable to assume that repeating acute PA may be required to preserve the positive PA-related effects on cognition over prolonged periods of SB (e.g., office working hours or esports training (Manci et al. 2024)). The precise time points to repeat acute PA may be conceptualized by the PA density construct.

That density can be an important variable for informing the prescription of PA is also supported by several lines of evidence extending from the behavioral level to the molecular and cellular levels and functional brain level. In particular, two studies in healthy younger adults investigated the effects of two repeated acute bouts of high-intensity interval exercise (HIIE, 4 × 4-min work bouts at 90% of VO_2_ peak interspersed with 3-min rest bouts at 60% VO_2_ peak) on inhibitory control (i.e., assessed by the Stroop task every 10 min after the cessation of each bout of physical exercise for 5× times) (Hashimoto et al. 2018; Tsukamoto et al. 2016b). In both studies, a recovery interval of 60 min separated the first bout of acute HIIE from the second bout of HIIE, in which the Stroop task performance was repeatedly assessed (Hashimoto et al. 2018; Tsukamoto et al. 2016b). These studies showed that inhibitory control (i.e., reverse Stroop interference score) improved immediately (Hashimoto et al. 2018; Tsukamoto et al. 2016b) and 10 min (Tsukamoto et al. 2016b) after exercise cessation in both the first and second bout acute bouts of HIIE compared to the pretest. For the first acute bout of HIIE an inhibitory control benefit persisted for up to 40 min following exercise cessation (Hashimoto et al. 2018; Tsukamoto et al. 2016b). For the second bout of HIIE the change in inhibitory control at the 10 (Hashimoto et al. 2018) or 20 min (Tsukamoto et al. 2016b) assessment interval after exercise cessation was not significantly different from the pretest and was less pronounced compared to the first bout of HIIE when considering the post-exercise assessments at 20 (Hashimoto et al. 2018), 30 (Hashimoto et al. 2018), and 40 (Hashimoto et al. 2018; Tsukamoto et al. 2016b) but not 50 min (Hashimoto et al. 2018; Tsukamoto et al. 2016b).

Collectively, these observations suggest that the acute PA-related effects on inhibitory control were less pronounced in the second bout of HIIE compared to the first bout of HIIE. Hypothetically, such a diminished effect after the second bout of HIIE could be, among other factors, related to the relatively close temporal proximity (i.e., high density) between the two single bouts of HIIE (i.e., 60 min).

In addition to examining postexercise inhibitory control, the work described in the previous paragraph observed that acute PA-induced performance improvements in inhibitory control correlated with changes in blood lactate concentration in both studies (Hashimoto et al. 2018; Tsukamoto et al. 2016b), and that changes in peripheral blood lactate concentration were significantly lower during and after the second bout of HIIE (Tsukamoto et al. 2016b). Given these results, it seems reasonable to speculate that there is a neurobehavioral relationship between each measure (Hashimoto et al. 2021; Herold et al. 2019a, b; Törpel et al. 2018; Yamada et al. 2021) and is an assumption supported by evidence that peripheral blood lactate can cross the blood–brain barrier via monocarboxylate transporters and be utilized as “fuel” for cognitive processes (Brooks 2018, 2020a, b; Brooks et al. 2021, 2022, 2023; Quistorff et al. 2008; Riske et al. 2017; Taher et al. 2016). Indeed, recent studies have reported that changes in peripheral blood lactate concentration are correlated with acute PA-related improvements in cognitive performance (Ballester-Ferrer et al. 2022; Herold et al. 2022a; Nunes Pereira Oliva et al. 2023); however, it remains unclear whether blood lactate changes are a mediator of acute PA-induced benefits on cognitive performance (Li et al. 2023). In addition, there is evidence that a change in peripheral blood lactate concentration (e.g., induced by acute physical exercise (Ferris et al. 2007) or infusion at rest (Schiffer et al. 2011) is associated with a change in the concentration of serum levels of the brain-derived neurotrophic factor (BDNF), an important neurotrophin involved in processes of PA-related neuroplasticity and brain health (Cefis et al. 2023; Erickson et al. 2010, 2011; Knaepen et al. 2010; Leckie et al. 2014; Marston et al. 2019; Stimpson et al. 2018; Walsh et al. 2020; Walsh and Tschakovsky 2018).

In younger healthy adults, acute PA-induced changes in the BDNF are correlated with cognitive improvements (Hwang et al. 2016), lending credence to the hypothesis that BDNF is involved in cognitive performance improvements after an acute bout of PA (Borror 2017). Such acute PA-triggered effects of BDNF on measures of cognitive performance are transient, as several studies on the kinetics of BDNF have consistently shown that elevated BDNF levels return to baseline 15–60 min after exercise cessation (for review, see (Dinoff et al. 2017)), and is a result that may explain, among other factors, why the after-effects of acute PA on cognitive performance lasting for a limited time. Accounting for the transient nature of the “facilitation effect”, which is characterized by the acute PA-induced release of neurotrophic factors such as BDNF (Herold et al. 2018a; Fissler et al. 2013), by the PA variable density is highly relevant for specific types of PA interventions (e.g., sequential motor-cognitive training). Concerning motor-cognitive interventions, considering acute density would allow for better standardization of the time at which prolonged periods of cognitive exercise need to be interrupted by acute PA to ensure an optimal facilitation effect across the entire training session (Herold et al. 2018a).

The transient effects of acute PA are not only evinced at the behavioral, or molecular and cellular levels but also at the functional brain level (e.g., cerebral blood flow [CBF]), which is hypothesized to mediate the acute effects of PA on cognitive performance (Pontifex et al. 2019). Indeed, some studies provide evidence that acute PA-induced changes in cerebral artery velocity (CBV), a surrogate for CBF that is typically measured by monitoring middle CBV via transcranial Doppler ultrasound (Ide and Secher 2000; Kennedy et al. 2022; Mulser and Moreau 2023; Tymko et al. 2018), correlate with acute PA-induced cognitive improvements (i.e., executive functioning assessed by the antisaccade task) (Shirzad et al. 2022; Tari et al. 2020). The acute PA-induced increase in CBV can persist for up to 2 h after exercise cessation depending on several factors (e.g., characteristics of the person and the acute bout of PA, methodological factors—for review see (Kennedy et al. 2022)) but typically return to baseline levels relatively shortly after exercise cessation (Mulser and Moreau 2023; Kennedy et al. 2022) (e.g., 30 min—for review see (Kennedy et al. 2022)) underpinning the transient nature of PA-induced effects on putative neurobiological mechanisms which may mediate the effects of acute PA on behavioral measures of brain health.

Taken together, the current state of evidence supports the view that acute PA-related changes may be transient although the exact time course (e.g., precise time point to harvest the largest after-effects of acute PA) and potential moderators of the after-effects of acute PA (e.g., acute PA-related factors such as type, intensity, duration, and non-PA-related factors such as age, biological sex, health status, and fitness level) on specific measures of brain health are not fully understood and is — in part — related to methodological challenges (e.g., a limited number of follow-up assessments, confounding influence of activities performed between cessation of acute PA and cognitive test administration) (Pontifex et al. 2019). However, the evidence on the after-effects of acute PA urges future research to consider acute PA density as a variable to facilitate our understanding of the dose–response relationship between acute PA and brain health. In particular, considering the after-effects of acute PA on different levels of analysis can help to inform the prescription of PA density, which, in turn, allows for the standardized prescription of the time point(s) at which the acute PA stimulus needs to be repeated to prolong the acute PA-related benefits on specific measures of brain health (e.g., time points to interrupt prolonged sitting during sedentary working activities or cognitive exercise in sequential motor-cognitive interventions). Such information on the appropriate timing to set a PA stimulus is crucial to inform the experimental design of studies (e.g., studies investigating the effects of breaking up prolonged sitting by acute PA on brain health (Chueh et al. 2022; Li et al. 2022)) and to maximize the effectiveness of PA interventions (e.g., “just-in-time adaptive PA interventions” (Hardeman et al. 2019; Müller et al. 2017; Wunsch et al. 2022) or interventions with sequential motor-cognitive exercises (Herold et al. 2018a)).

**Fig. 2:**
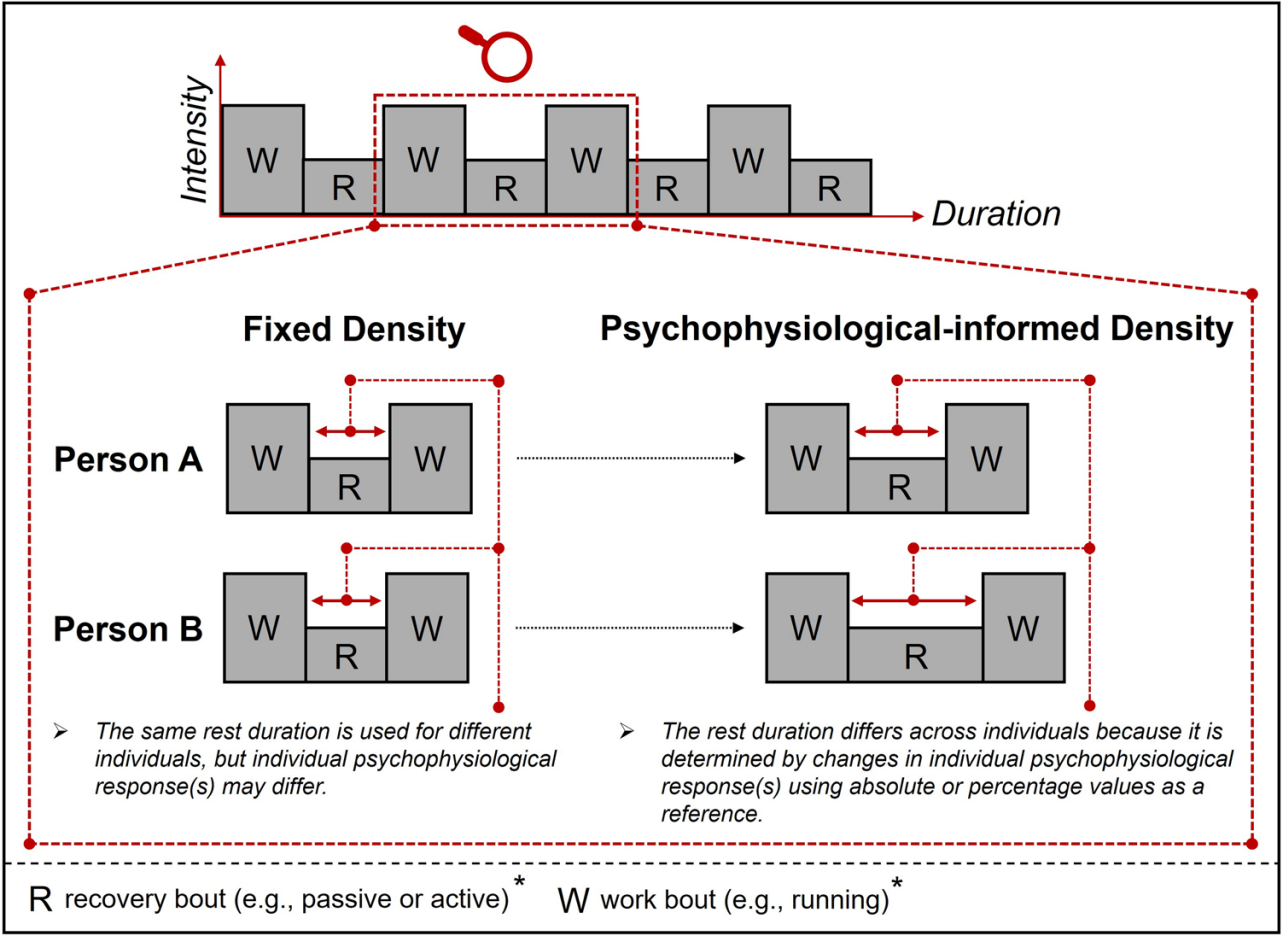
Schematic illustration of the conceptual differences between (1) *fixed density* and (2) *psychophysiological-informed density* that can be used to prescribe a specific (acute) density of physical activity (Manci et al. 2024). Based on recent work, we propose that in addition to using traditional psychophysiological parameters (e.g., heart rate; Buchheit and Laursen 2013b) to prescribe acute density in planned and structured forms of physical activity, changes in pupil size (Matsui et al. 2024) and brain hemodynamics (Herold et al. 2020b) can be promising psychophysiological parameters to inform an acute, brain health-centered, psychophysiological-informed density prescription (e.g., using a fixed value or percentage of a reference value; Buchheit and Laursen 2013b). The asterisk (*) indicates that other acute (i.e., type of physical activity, intensity, and acute duration) and chronic variables (i.e., chronic duration) that characterize the bout(s) of physical activity are assumed to be constant

As shown in Fig. 2, we propose two different approaches, namely (1) “*fixed acute PA density*” in which the time intervals are fixed for all individuals (e.g., breaking up prolonged sitting of 8 h every 30 min by acute PA), and (2) “*psychophysiological-informed acute PA density*” in which the time intervals are informed by actual changes in specific psychophysiological markers (e.g., comparable to the approach to use psychophysiological changes such as in CBF (Herold et al. 2020b) or affective response (Baldwin et al. 2016; Ladwig et al. 2017; Parfitt et al. 2012; Zenko et al. 2024) for exercise intensity prescription) to set and study the influence of acute PA density (Manci et al. 2024). For example, a “psychophysiological-informed acute PA density” approach might use changes in physiological markers such as pupil size (i.e., a decrease of pupil size below a specific threshold) to determine the exact duration at which periods of prolonged sitting should be interrupted by acute PA – as highlighted for a specific application scenario (i.e., esports) elsewhere (Manci et al. 2024).

### Chronic density—simple analysis approaches

In observational or intervention studies, an approach to investigate the influence of different chronic density patterns on brain health is to study different groups of individuals based on their chronic PA density patterns (e.g., a low chronic PA density group in which individuals perform PA on non-consecutive days versus a high chronic PA density group in which individuals perform PA on consecutive days—see also Fig. 1 b).

### Chronic density—sophisticated analysis approaches

The use of more sophisticated approaches, including distributional data analysis (Ghosal and Matabuena 2024; Ghosal et al. 2022), compositional isotemporal data analysis (Bezerra et al. 2021; Dumuid et al. 2019, 2020; Hyodo et al. 2022; Lau et al. 2023; Lu et al. 2023; Mitchell et al. 2023), burstiness analysis (Culverhouse et al. 2024; Takeuchi and Sano 2024), and machine learning (Memel et al. 2016; Poudel et al. 2022), holds promise for identifying groups of individuals with distinct chronic PA density patterns. Despite some limitations and challenges (e.g., the need for large sample sizes and high-dimensional data, the time-consuming nature of training algorithms, and the lack of benchmark data), machine learning-based approaches in particular provide several advantages for the purpose of profiling PA patterns (e.g., more accurate classification and prediction, the possibility of a hypothesis-free/generating approach) (Clark et al. 2021; Farrahi and Clare 2024; Farrahi et al. 2019; Farrahi and Rostami 2024; Fuller et al. 2022). Another advantage of machine learning-based approaches is their capacity to handle large, complex, and high-dimensional datasets (Farrahi and Rostami 2024). The ability and flexibility to handle such datasets make machine learning-based approaches well-suited for analyzing the influence of  PA density on specific markers of brain health because density is a more complex variable than other PA variables (e.g., frequency, duration). This assumption is supported by the fact that these approaches have been successfully applied to elucidate the influence of “micropatterns” of PA including intensity and duration (also referred to as bout length) on health-related outcomes such as mortality (Ahmadi et al. 2023; Stamatakis et al. 2022) and cancer incidence (Stamatakis et al. 2023). Thus, extending machine learning-based approaches to density is a promising area for future research to elucidate the influence of different chronic PA density patterns on measures of health in general and brain health in particular.

The application of sophisticated classification and analysis techniques may enable the investigation of specific research questions (e.g., *Is a low density of moderate-intensity PA in older adults more, less, or equally beneficial for brain health than having a high density of moderate-intensity PA?)* or to study the association of specific density-related PA patterns, such as the regularity and randomness of PA density, with measures of brain health. In this context, we propose that the regularity and its antithesis randomness can be characterized by the periodicity and the stability (Shi et al. 2024) that is in the case of the PA density reflected by the degree of variability of the duration of the rest bouts between successive work bouts within a given time interval (e.g., day, week, month, year). We suggest that, among other approaches (Rowlands et al. 2015), the regularity and randomness of PA density can be operationalized by further developing different measures used to assess fragmentation (Gao et al. 2023; Danilevicz et al. 2024a, b; Marino et al. 2024, 2025; Wanigatunga et al. 2022), complexity and fractal dynamics (Blodgett et al. 2023; Cavanaugh et al. 2010; Hu et al. 2016; Raichlen et al. 2019), or entropy (Shi et al. 2024).

Fragmentation of the RAR is typically assessed using metrics (for an overview see (Gao et al. 2023)) such as intra-daily variability (IV) and inter-daily stability (IS), providing information on how constant RAR is within or across days (Danilevicz et al. 2024b, 2024a; Gao et al. 2023; Vetter 2020), or transition probability (TP), a measure quantifying the likelihood of transitioning from rest (e.g., sleep) to activity (e.g., PA), or vice versa (Danilevicz et al. 2024b, 2024a; Lim et al. 2011; Marino et al. 2025).

Concerning IV of the RAR, there is evidence that (1) older adults with early-onset dementia (Hooghiemstra et al. 2015) or Alzheimer Disease (van Egroo et al. 2024) have a higher baseline IV of RAR compared to healthy controls, (2) in older adults with mild-moderate Alzheimer’s disease a higher baseline IV of RAR is associated with accelerated cognitive decline after a one-year follow-up (Targa et al. 2021), (3) in older adults a higher slope of IV of RAR is associated with stepper global cognitive decline (van Egroo et al. 2024), (4) in a sample of older adults with and without mild cognitive impairment (MCI) a lower baseline IV of RAR is associated with better executive functioning (Alfini et al. 2021), (5) in middle-aged and older adults a higher baseline IV of RAR is associated with higher risk for developing cognitive impairments (Haghayegh et al. 2024; Xiao et al. 2022) and dementia (Haghayegh et al. 2024), worse cognitive functioning (i.e. slower processing speed (Luik et al. 2015; Oosterman et al. 2009), worse executive functioning (Luik et al. 2015; Oosterman et al. 2009), worse memory (Oosterman et al. 2009), and worse global cognition (Luik et al. 2015)) as well as detrimental functional and structural brain changes (i.e., more pronounced amyloid deposit in the brain and cerebrospinal fluid (Musiek et al. 2018), lower temporal lobe volume (van Someren et al. 2019) and posterior parietal grey matter volume (Smagula et al. 2020), higher white matter lesion volume (Zuurbier et al. 2015), and the presence of cerebral microbleeds (Zuurbier et al. 2015)), (6) in a sample of older adults a higher baseline IV of RAR is associated with an increased risk of displaying hypopigmentation of the locus coeruleus, a marker of neurodegeneration, which relationship with cognitive performance (i.e., episodic memory, and global cognition) is mediated by IV of RAR (van Egroo et al. 2024), and (7) in a sample of middle-aged and older adults with subjective and/or objective cognitive impairment a higher baseline IV of RAR is associated lower cortical thickness in different cortical areas (i.e., right cuneus, left middle frontal gyrus, and lateral orbital frontal cortex) (Espinosa et al. 2025).

Regarding IS of RAR, there is evidence that (1) older adults with Alzheimer's Disease have a lower baseline IS compared to healthy controls (van Egroo et al. 2024), (2) in a sample of older adults with and without MCI, a higher baseline IS of RAR is associated with better episodic memory performance (Alfini et al. 2021), (3) in middle-aged and older adults, lower baseline IS of RAR is associated with worse cognitive performance (i.e., slower processing speed, worse executive functioning (Luik et al. 2015; Rabinowitz et al. 2022; Sun et al. 2023), worse memory performance (Sun et al. 2023), and faster memory decline (Rabinowitz et al. 2022)), as well as detrimental structural brain changes (e.g., more pronounced occipital periventricular and frontal white matter lesions (Oosterman et al. 2008), or a greater likelihood of white matter lesion burden in the anterior thalamic radiation (Palmer et al. 2022)), (4) in a sample of middle-aged to older adults with subjective and/or objective cognitive impairment a lower baseline IS of RAR with lower cortical thickness in frontal, temporal, and postcentral brain regions (i.e., left and right superior frontal gyrus, left superior temporal gyrus, and left postcentral gyrus) (Espinosa et al. 2025).

Concerning fragmentation (i.e., TP), an observational study reported that a lower PA fragmentation is associated with less cognitive decline in memory and visuospatial processing performance in cognitively unimpaired older adults (Marino et al. 2025).

Collectively, the evidence suggests that in middle-aged and older adults less fragmented, especially less variable RAR patterns (i.e., lower IV and higher IS of RAR) are associated with better brain health although additional, more rigorously designed examinations with larger and more diverse samples are warranted before more robust conclusions can be drawn (for review see also (Smagula et al. 2019; Zhang et al. 2024)). Whether such a finding is generalizable to patterns of chronic PA density needs to be elucidated in future studies, especially since another observational study using a different metric for fragmentation (i.e., PA variability quantified by the minute-to-minute PA changes across the observation period) reported that in a nationally representative sample of older adults less variable PA patterns are associated with higher odds of cognitive impairment, even after accounting for total PA and other demographic covariates such as age, sex, education, race/ethnicity, and health status (Donahue et al. 2025).

Fractal dynamics are characterized by self-affinity (also referred to as self-similarity or scale invariance) of a given signal (e.g., derived from accelerometers) across time scales (Arsac and Deschodt-Arsac 2018; Goldberger et al. 2002; Hardstone et al. 2012; Paraschiv-Ionescu et al. 2008; Pittman-Polletta et al. 2013). There is a strong case to be made that fractal dynamics can help to better understand the periodization of chronic physical exercise (Brown and Greenwood 2005), and several studies have used this approach to analyze physiological data (e.g., frequently applied to heart rate variability data (Gronwald et al. 2020a, 2021; Kaufmann et al. 2023; Rogers et al. 2021)), or PA patterns (Blodgett et al. 2023; Cavanaugh et al. 2010; Hu et al. 2016; Raichlen et al. 2019). In the context of PA, a popular method for assessing fractal dynamics (e.g., of PA (Blodgett et al. 2023; Cavanaugh et al. 2010; Hu et al. 2016; Raichlen et al. 2019)) is detrended fluctuation analysis (DFA), which is a nonstationary time-series analysis of specific signals (e.g., accelerometer data) that reflects the correlative structure and fractal dimension of signal fluctuations across a range of time scales based on a modified root-mean-square analysis (Hardstone et al. 2012; Peng et al. 1994, 1995a, 1995b; Pittman-Polletta et al. 2013). For example, a study using data from 5097 middle-aged adults showed that greater fractal stability of daily PA (i.e., assessed via a thigh-mounted accelerometer over seven days and reflected in a higher DFA scaling exponent) was associated with better verbal fluency performance in males but not in females (Blodgett et al. 2023). Such biological, sex-specific differences are consistent with the growing body of evidence suggesting that biological sex is an important moderator in the relationship between PA and brain health (Barha et al. 2020, 2017a, 2017c, 2019, 2023; Barha and Liu-Ambrose 2018, 2020). However, whether such findings extend to the chronic density of PA remains a promising area for further investigations.

### Recommendations regarding the assessment and prescription of chronic density

**Fig. 3:**
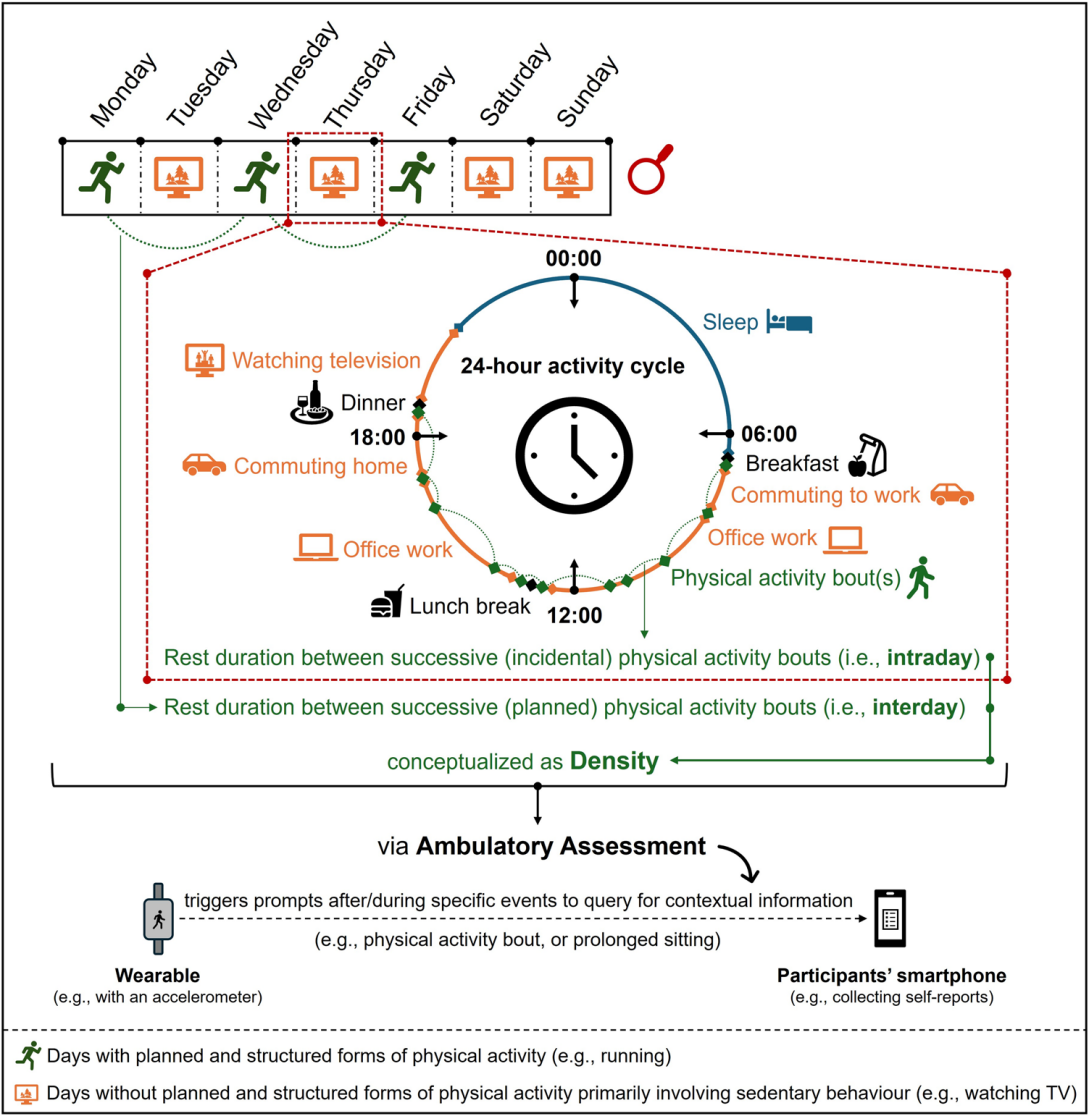
Schematic illustration of how the density of physical activity can be assessed considering narrower periods (i.e., a week and a day). Please note that the figure zooms in on a day without planned and structured physical activity (i.e., no physical exercise), but with incidental physical activity. The color code is as follows: (1) green color is related to physical activity, (2) orange color is related to sedentary behavior, and (3) blue color is related to sleep. In addition, we intentionally refrained from including other, less important, activities (e.g., showering, going to the toilet, talking to coworkers) in the 24-h activity cycle to maintain the clarity of the display item

To quantify the chronic density of PA, we recommend the application of device-based assessments to complement subjective assessments (i.e., questionnaires) for the following reasons. First, popular questionnaires to assess chronic PA, such as the International Physical Activity Questionnaire (IPAQ), quantify frequency but not chronic PA density (i.e., neither the long form (Craig et al. 2003) nor the short form (Lee et al. 2011) of the IPAQ), although some recently developed questionnaires do, at least in part, collect such information (e.g., Daily Activity Behaviours Questionnaire (Kastelic et al. 2023, 2021, 2022; Kastelic and Sarabon 2023)). Second, although subjective assessment tools (e.g., questionnaires) have several advantages (e.g., low burden for participants, cost-effective and convenient administration), they are prone to several sources of bias (e.g., recall bias or social desirability bias) that can confound the estimation of chronic PA patterns (Nigg et al. 2020; Prince et al. 2020; Strath et al. 2013; Warren et al. 2010). Hence, device-based assessment tools can circumvent the above-described limitations of subjective assessment tools; however, it should be considered that (1) the applied device-based measurement tool needs to be valid and reliable (Argent et al. 2022; Johnston et al. 2020; Mühlen et al. 2021), and (2) there is not yet a fully established consensus on the application of device-based measurement tools (e.g., placement and sampling frequency of the device) or on the data processing procedures to obtain specific indices of PA (e.g., minimal length of the epochs, filter, cut-off points, non-wear-time definition) although some recommendations exist (Migueles et al. 2021, 2017; Pulsford et al. 2023; Rodrigues et al. 2025). Further, we recommend combining popular device-based tools such as accelerometers with other sensors (e.g., for ambient light, barometer/altimeter, or geolocation) and digital tools (e.g., smartphones) to allow for the recording of contextual information (e.g., weather via geolocation at a specific time point (Timm et al. 2023) or type of activity conducted during rest bout(s) via an accelerometer-triggered electronic-diary (Ebner-Priemer et al. 2013; Giurgiu et al. 2019, 2020a, 2020b, 2020c; Reichert et al. 2020; Timm et al. 2023)). The latter approach is also referred to as ambulatory assessment (see Fig. 3), which typically also includes the assessment of the context in which behaviors occur (Haaren-Mack et al. 2022; Reichert et al. 2020; Trull and Ebner-Priemer 2013).

Regarding PA density, rest bouts are a key construct and may be considered synonymous with, or primary to, the time spent in SB when considering waking hours. When accounting for non-waking hours, rest encompasses time spent sleeping. As we will demonstrate below, SB and sleep, are lifestyle behaviors that can moderate the effect of PA on brain health (Collins et al. 2023; Mellow et al. 2019, 2022). Thus, they should be considered important moderators when aiming to elucidate the effect of PA density on brain health, especially when studying chronic PA.

Concerning SB, there is emerging evidence that the effect of acute PA on cognitive function is altered by subsequent exposure to prolonged sitting versus breaks in sitting (Wheeler et al. 2019), and that the characteristics of activities that are primarily involved in the rest bout(s) can differentially influence brain health (Hallgren et al. 2020; Mallawaarachchi et al. 2024; Raichlen et al. 2022; Zou et al. 2024). In particular, there is evidence that the type of SB can moderate the effects of SB on brain health because mentally active SB (e.g., reading or working on a computer) are often positively associated with brain health, whereas mentally passive SB (e.g., watching non-educational television programs) does not confer such benefits (Hallgren et al. 2020; Mallawaarachchi et al. 2024; Raichlen et al. 2022; Zou et al. 2024). In addition, a growing body of evidence suggests that the brain and cardiometabolic consequences of too much time spent in SB are distinct from those of too little PA (Dunstan et al. 2021) (Pinto et al. 2023; Raichlen et al. 2023; Zou et al. 2024). This reinforces the utility of considering SB as a mechanism for the importance of density as a key novel element to complement the FITT and FITT-VP principles.

Concerning sleep (i.e., often quantified as time in bed), there is accumulating evidence that it can mediate and/or moderate the effect of PA on brain health (Bloomberg et al. 2023; Liu-Ambrose and Falck 2019; Mellow et al. 2019, 2022). For example, several cross-sectional studies provide evidence that (1) subjective sleep quality and sleep efficiency mediate the relationship between PA level and inhibitory control in younger adults (Li et al. 2021), (2) sleep efficiency mediates the relationship between PA and working memory, task switching, verbal ability and fluency, and memory recall in a mixed sample of younger and older adults (Wilckens et al. 2020), (3) better subjective sleep quality mediates the relationship between PA and verbal fluency, immediate recall, delayed recall (Cheval et al. 2022) and working memory (Guardia et al. 2024) in middle-aged and older adults, and (4) older adults with poor sleep efficiency (i.e., percent of the time in bed spent asleep) benefit most from PA in terms of global cognition (Callow et al. 2024). As well, a 6-month intervention study wherein cognitively healthy older adults performed moderate- or vigorous-intensity interval exercise twice a week, reported that participants in the moderate-intensity group with documented poorer baseline sleep efficiency showed a greater exercise-induced improvement in episodic memory and global cognition (Sewell et al. 2023).

In conjunction with the observations that other activities of the 24-h cycle, which can form rest bout(s), including free-living standing activity (Halloway et al. 2021) and light-intensity PA (Erlenbach et al. 2021) are positively associated with cognitive performance, the above-summarized evidence on the moderating role of SB and sleep supports the idea that considering all activities in the 24-h activity cycle is important to advance our understanding of the influence of PA on brain health (Mellow et al. 2022, 2019; Collins et al. 2023). To this end, complementing the 24-h activity approach with PA density may enable more nuanced insights into the complex relationships between lifestyle behaviors and brain health (see Fig. 3).

## Limitations

Using brain health as the case in point, we have discussed the opportunities and challenges for further research employing the variable *density* to improve the understanding of dose–response relationships between PA and brain health; however, the following limitations need to be considered.

First, others have previously advocated for complementing FITT from a psychological perspective. In this view, an additional “F” representing “fun”, an umbrella term for psychological factors such as affective valence and enjoyment of PA (Burnet et al. 2018), is used to reflect that these factors are important determinants of PA engagement and adherence (Brand and Cheval 2019; Brand and Ekkekakis 2018, 2021; Cheval and Boisgontier 2021; Collado-Mateo et al. 2021; Ekkekakis and Brand 2019).

Second, specific populations (e.g., individuals with chronic diseases), depending on other exercise and training variables (e.g., exercise intensity and training frequency) that determine the overall PA dosage, may not well-tolerate PA with a relatively high acute or chronic PA density. Thus, to minimize the risk of PA-related adverse health events in such special populations (e.g., individuals with eating disorders, or older adults without/with cognitive impairment), they may require a personalized PA prescription, which especially considers adapting PA density based on the individual PA capacity and tolerance.

Third, although we provide a strong theoretical rationale that complementing FITT and FITT-VP with the variable density will improve our understanding of the dose–response relationship between PA and brain health, we wish to emphasize that the precise characterization or prescription of a specific PA dosage will remain a considerable challenge because of the myriad of (1) non-modifiable factors (e.g., age, biological sex, genetics), (2) potentially modifiable non-PA-related factors (e.g., diet, sleep, stress, environmental conditions), and (3) modifiable PA-related factors (e.g., PA type, intensity, duration, movement frequency), which include but are not limited to setting (e.g., home-based or center-based, and indoor or outdoor), method of delivery (e.g., in-person or online), level of supervision (e.g., no supervision, general supervision, direct supervision) and social interaction (e.g., individual or group-based), that can influence the dosage and individual psychophysiological response(s) to PA, as discussed elsewhere in more detail (Barha et al. 2017a, b, 2019, 2021a, b; Barha and Liu-Ambrose 2020, 2018; Gronwald et al. 2020b; Herold et al. 2019a, 2024a, b; Meyler et al. 2021; Solis-Urra et al. 2024; Toigo and Boutellier 2006). In other words, adding density to FITT and FITT-VP is another piece of the puzzle to improve the determination of the PA dosage and, in turn, disentangle its influence on specific health-related outcomes such as brain health.

## Conclusions

Density, a variable that characterizes the timing of PA within a specific time period; it does so by quantifying the temporal intervals (i.e. time spent resting) between successive working bouts within a single PA bout as well as between successive PA bouts within a specific time period, has been under-recognized in the contemporary scientific practice when studying the relationship between PA and health-related outcomes. Using the field of brain health as an example, this article has provided an overview of the implications and the potential of addressing PA density as a variable that gives an additional piece of information that complements traditional concepts (i.e., FITT and FITT-VP) for PA dosage determination and allows for more precise prescription of it for improved health effects and the prevention and treatment of chronic disease. Considering that (1) there is an increasing interest in understanding the health effects of PA dosage, including but not limited to “micropatterns” of PA typically assessed via high-resolution data of wearables (Ahmadi et al. 2023; Gill et al. 2023; Stamatakis et al. 2022), and (2) an explicit focus on the density variable has been largely absent from PA research to date, we discussed approaches for operationalizing PA density and showing how investing greater effort in understanding its effects can add fruitful nuance to identifying the dose–response relationship between PA and health-related outcomes (e.g., brain health), and thus has the potential to provide important information on the optimal and minimal beneficial PA dosage.

## Data Availability

Data sharing does not apply to this article because no data were created or analyzed for this review article.
